# Predicting Writing Styles of Web-Based Materials for Children’s Health Education Using the Selection of Semantic Features: Machine Learning Approach

**DOI:** 10.2196/30115

**Published:** 2021-07-22

**Authors:** Wenxiu Xie, Meng Ji, Yanmeng Liu, Tianyong Hao, Chi-Yin Chow

**Affiliations:** 1 Department of Computer Science City University of Hong Kong Hong Kong China (Hong Kong); 2 School of Languages and Cultures The University of Sydney Sydney Australia; 3 School of Computer Science South China Normal University Guangzhou China

**Keywords:** online health education, health educational resource development, machine learning, health linguistics

## Abstract

**Background:**

Medical writing styles can have an impact on the understandability of health educational resources. Amid current web-based health information research, there is a dearth of research-based evidence that demonstrates what constitutes the best practice of the development of web-based health resources on children’s health promotion and education.

**Objective:**

Using authoritative and highly influential web-based children’s health educational resources from the Nemours Foundation, the largest not-for-profit organization promoting children’s health and well-being, we aimed to develop machine learning algorithms to discriminate and predict the writing styles of health educational resources on children versus adult health promotion using a variety of health educational resources aimed at the general public.

**Methods:**

The selection of natural language features as predicator variables of algorithms went through initial automatic feature selection using ridge classifier, support vector machine, extreme gradient boost tree, and recursive feature elimination followed by revision by education experts. We compared algorithms using the automatically selected (n=19) and linguistically enhanced (n=20) feature sets, using the initial feature set (n=115) as the baseline.

**Results:**

Using five-fold cross-validation, compared with the baseline (115 features), the Gaussian Naive Bayes model (20 features) achieved statistically higher mean sensitivity (*P*=.02; 95% CI −0.016 to 0.1929), mean specificity (*P*=.02; 95% CI −0.016 to 0.199), mean area under the receiver operating characteristic curve (*P*=.02; 95% CI −0.007 to 0.140), and mean macro F1 (*P*=.006; 95% CI 0.016-0.167). The statistically improved performance of the final model (20 features) is in contrast to the statistically insignificant changes between the original feature set (n=115) and the automatically selected features (n=19): mean sensitivity (*P*=.13; 95% CI −0.1699 to 0.0681), mean specificity (*P*=.10; 95% CI −0.1389 to 0.4017), mean area under the receiver operating characteristic curve (*P*=.008; 95% CI 0.0059-0.1126), and mean macro F1 (*P*=.98; 95% CI −0.0555 to 0.0548). This demonstrates the importance and effectiveness of combining automatic feature selection and expert-based linguistic revision to develop the most effective machine learning algorithms from high-dimensional data sets.

**Conclusions:**

We developed new evaluation tools for the discrimination and prediction of writing styles of web-based health resources for children’s health education and promotion among parents and caregivers of children. User-adaptive automatic assessment of web-based health content holds great promise for distant and remote health education among young readers. Our study leveraged the precision and adaptability of machine learning algorithms and insights from health linguistics to help advance this significant yet understudied area of research.

## Introduction

### Background

Web-based health education and promotion has become increasingly popular among all age groups [1]. Although existing research on web-based health educational materials has focused on adults or general readers, there is an increasing body of research on the assessment and evaluation of web-based educational resources on children’s health [2,3]. Clinical and academic research shows that effective writing styles can have an impact on the understanding and reception of medical and health educational resources for different reader groups [4-6]. There is a pressing need to investigate the writing style of web-based health resources on children’s health promotion and education for the main readers of such materials as parents and child caregivers to ensure information relevance and acceptability. The Agency for Healthcare Research and Quality is the lead federal agency charged with improving the safety and quality of America’s health care system, including pediatric health care products and services [7]. The Agency for Healthcare Research and Quality has developed the Patient Education Materials Assessment Tool (PEMAT) to ensure the development and delivery of quality health care products and services. Key assessment criteria of PEMAT include health information understandability, relevance, and actionability [8,9].

Much of the current research has focused on exploring these assessment dimensions separately using long-standing readability tools [10-13] or machine learning algorithms of natural language features [14-16] using features such as general medical vocabularies, consumer medical vocabulary, natural language features such as a part of speech features [17-19], and other metadata [20]. Furthermore, many of these data-intensive and data-driven studies did not consider insights from research fields directly relevant to health educational resource development and evaluation. The lack of model interpretability has largely limited the applicability of such computational research in practical health education. How to effectively link linguistic research, health education, and machine learning modeling needs to be addressed.

The core question of our study is to develop machine learning models to discriminate and predict what constitutes a suitable writing style of web-based health resources on children’s health promotion and education. Research-based evidence is needed to inform and improve the current practice of web-based health educational resource development on health issues related to the promotion of children’s health and well-being for readers such as parents, caregivers of children, and teenagers. Our study aims to assess the writing styles of web-based health resources on children’s health through an integrated, holistic approach, that is, the development of machine learning models to evaluate whether the content and the writing style of a piece of web-based health educational material is more related to children’s health promotion and education, or more for the general public. The underlying hypothesis of our study is that the content and writing style of high-quality web-based health educational resources vary with the intended readership, which is based on the principles of clinically developed guidelines for health educational resource assessment such as PEMAT [21-23] and health educational research findings in support of user-oriented health communication styles [24-31].

### Data Sets and Feature Extraction

#### Corpus Data Collection and Classification

The Nemours Foundation is the world’s largest nonprofit organization dedicated to improving the health and well-being of children, and the website of the Foundation has high-quality health education resources developed by medical experts and experienced health educators purposefully for different readerships including parents, children (aged ≤13 years) and teenagers (aged 13-20 years) [32]. Given the inherent difficulties of conducting large-scale surveys of web-based health educational materials among young children, we used high-quality, authoritative, and children-oriented health materials on the KidsHealth website [33] as the training data to develop machine learning algorithms to predict the relevance and suitability of health education resources for young children with English as the native language. The entire data set contains around 200 children-oriented health texts and 800 adult health texts that we collected on websites developed by nonprofit health organizations and intended for the public, such as the World Health Organization (Multimedia Appendix 1 presents some of the websites used).

#### Text Screening Criterion

For the selection of health information for the general public, the main screening criteria were that the websites must have been certified by the Health on the Net Foundation, an international accreditation authority of web-based health information, and they must have been developed by health authorities to provide accurate health educational information. These included governmental health organizations, accredited nonprofit health organizations engaged in health promotion and education, or national or regional associations of specific disease prevention and control. We carefully screened a total of 200 children’s health readings from the website of Nemours KidsHealth [33] as one of the most authoritative children health education websites, accredited by the Health on the Net Foundation [34] for its authority (details of the editorial team and the site team are clearly stated), justifiability (health information is complete and provided in an objective, balanced, and transparent manner), and transparency (the site is easy to use, and its mission is clear). The intended readers were clearly the parents and caregivers of children, as shown in the user-specific website structure. It should be noted that there was a clear imbalance between the two sets of health texts, which reflects the reality of web-based health educational resources, as children-oriented health materials are much less than adult-oriented health resources.

#### Corpus Annotation of Semantic Features

We annotated the health texts using the semantic tagging system developed by the University of Lancaster, United Kingdom [35]. The annotated health texts contained 115 semantic features under 21 lexical categories—A: general or abstract terms; B: the body and individual; C: arts and crafts; E: emotions; F: food and farming; G: government and politics; H: architecture, housing, and the home; I: money, commerce, and industry; K: entertainment, sports, or games; L: live and living things; M: movement, location, travel, and transport; N: numbers and measurement; O: substances, materials, objects, and equipment; P: education; Q: language or communication; S: social actions, states, and process; T: time, W: world and environment; X: psychological actions, states, and processes; Y: science or technology; Z: names and grammars. Although the University of Lancaster Semantic Annotation System (USAS) was developed for general English studies, it has wide applications in specialist language studies, including health education and information. It is one of the most commonly used English semantic annotation systems.

Our study chose USAS purposefully, as we aimed to select linguistic and semantic features that may be used for developing machine learning algorithms to predict the semantic relevance and suitability of web-based health information among children. The semantic features described earlier are more suitable for analyzing and modeling the content relevance of health information. Many current studies use grammatical or syntactic features to develop machine learning algorithms for health information evaluation. However, grammatical, syntactic, morphological, or other types of structural or functional linguistic features cannot be used to study the contents of health information. The relevance of health information content for specific populations is largely underexplored in current health informatics using natural language processing and machine learning. Our study took advantage of the extensive English semantic coverage of USAS and developed algorithms using a small number of semantic features (20 from the original 115 semantic features) that measured diverse dimensions of the relevance and suitability of web-based health contents for English-speaking young children: approaches to medical knowledge acquisition; assessment of health situations; describing efforts; complexity of actions; attention, stress, or emphasis on key points; and finally, communicative interactivity. All these dimensions of health information relevance and suitability for young readers are supported and represented by semantic features incorporated in the comprehensive annotation system of USAS.

#### Statistical Analysis

Table 1 shows the Mann-Whitney *U* test of linguistic features as statistically significant features in web-based health education texts on the education of children’s versus adults’ health. The results show that children-oriented and adult-oriented health resources had statistically significant differences in the originally annotated semantic features (n=115). In addition to the two-tailed *P* values, the effect sizes (Cohen *d*) of the independent sample two-tailed *t* test were produced to measure the statistical differences between the two sets of health texts. As the mean differences were taken between health texts for children and adult health promotion, a positive Cohen *d* effect size indicated that a certain semantic feature is a characteristic feature of children-oriented health resources. A negative Cohen *d* effect size suggested that a semantic feature is more significant in health educational resources intended for the public.

A number of semantic features were identified as characteristic of adult-oriented health resources: semantic features that had large negative Cohen *d* effect sizes (above 0.5) included B2 health and disease (*P*<.001; Cohen *d*=−0.802); B3 medicine and medical treatment (*P*<.001; Cohen *d*=−0.800); Z2 geographical names (*P*<.001; Cohen *d*=−0.674); Z3 other proper names (*P*<.001; Cohen *d*=−0.594); M7 places (*P*<.001; Cohen *d*=−0.587); Y1 science and technology generally (*P*<.001; Cohen *d*=−0.522); Z99 out-of-dictionary rare expressions (*P*lt;.001; Cohen *d*=−0.776); A15 safety or danger (*P*<.001; Cohen *d*=−0.543); and S1 social actions, states, and processes (*P*<.001; Cohen *d*=−0.547). Semantic features with medium effect sizes (Cohen *d*=−0.5 to 0.3) were related to social processes, money, religion, and numeracy: G1 government, politics, and election (*P*<.001; Cohen *d*=−0.496); W3 geographical terms (*P*<.001; Cohen *d*=−0.414); L1 life and living things (*P*<.001; Cohen *d*=−0.391); I1 money generally (*P*<.001; Cohen *d*=−0.370); L2 living creature (*P*<.001; Cohen *d*=−0.362); S5 social groups and affiliation (*P*<.001; Cohen *d*=−0.356); S9 religion (*P*=.001; Cohen *d*=−0.324); and N1 numbers (*P*=.006; Cohen *d*=−0.315).

Textual features that were statistically significant in children-oriented health texts reflected the different cognitive processing of health information and health communication styles between children and adults. Semantic features that had a large Cohen *d* effect size (0.5-0.9) for children-oriented health texts included words indicating simple actions and steps: M1 moving, coming, and going (*P*<.001; Cohen *d*=0.547); M2 putting, taking pulling, and pushing (*P*<.001; Cohen *d*=0.517); E2 emotional expressions of like or dislike feelings (*P*<.001; Cohen *d*=0.556); X3 sensory words describing sight, taste, feel, and touch feelings (*P*<.001; Cohen *d*=0.684); S4 kinships (*P*<.001; Cohen *d*=0.713); X8 expressions describing efforts, attempts, and resolution (*P*<.001; Cohen *d*=0.803); and words of textual coherence or logical structure—Z8 pronouns (*P*<.001; Cohen *d*=0.907); Z6 negative expression (*P*<.001; Cohen *d*=0.764); and Z7 conditional expressions (*P*<.001; Cohen *d*=0.575).

There were two semantic categories related to emphasis, stress, and attention: A14 focusing subjuncts that draw attention to or focus on (*P*=.04; Cohen *d*=0.519) and A13 words as maximizers, boosters, approximators, and compromisers (*P*<.001; Cohen *d*=0.645). Semantic features that were identified as characteristic features of children-oriented health reading of a medium Cohen *d* effect size (0.3-0.5) included F1 food-related expressions (*P*<.001; Cohen *d*=0.493); O1 substances and materials generally (*P*<.001; Cohen *d*=0.49); B1 terms relating to the human body and bodily processes (*P*=.002; Cohen *d*=0.362); O4 physical attributes (*P*<.001; Cohen *d*=0.348); and E4 expressions of happiness or sadness (*P*<.001; Cohen *d*=0.493).

The large number of semantic features of statistical significance (*P*<.05) and medium-to-large effect sizes (Cohen *d* 0.3-0.9) needed to be further reduced to a smaller set of textual features to ensure the stability, efficiency, and convenience of any empirical assessment tool to be developed. The following sections will elaborate on machine learning–assisted automatic feature selection, followed by a review and revision of the empirical analytical instrument from the perspective of user-adaptive health resource design and health linguistics. The final machine learning model aims to provide high-precision automated predictions of the suitability of web-based health educational resources for young readers.

Machine learning algorithms are known for their lack of interpretability compared with statistical models. Through the successive permutation of the predictor features in the final algorithm (Gaussian Naive Bayes [GNB]), we calculated the impact of individual features on the performance of the algorithm, that is, its sensitivity and specificity. Two sets of semantic features were identified as significant contributors to the prediction of children- versus adult-oriented health educational resources. Each set of features that emerged in the process of algorithm development represented a balanced combination of semantic classes, which were statistically significant features in children- or adult-oriented materials.

**Table 1:** Semantic feature of health educational texts.

Semantic features	Children-oriented, mean (SD)	Adult-oriented, mean (SD)	Statistical difference		Effect size (Cohen *d*)
			Mann-Whitney *U* test	*P* value^a^	
A5: evaluation: good or bad	5.65 (7.267)	4.1 (4.994)	67510.0	.17	0.340
A15: safety or danger	0.230 (1.020)	1.560 (3.950)	56287.0	<.001	−0.543
B2: health and disease	7.910 (13.792)	22.45 (30.619)	41001.0	<.001	−0.802
B3: medicine and medical treatment	4.360 (8.392)	12.46 (17.280)	46443.5	<.001	−0.800
F1: food	10.30 (25.407)	3.490 (13.801)	51368.0	<.001	0.491
M1: moving, coming, going	5.27 (7.399)	2.92 (5.259)	52775.0	<.001	0.547
S1: social actions, states, and processes	1.850 (2.738)	3.820 (6.090)	54876.5	<.001	−0.547
S2: people	12.42 (15.635)	10.22 (16.519)	65131.5	.04	0.207
S4: kin	2.860 (4.221)	1.070 (3.247)	52886.5	<.001	0.713
S5: groups and affiliation	1.500 (3.672)	2.520 (4.771)	58355.0	<.001	−0.356
S8: helping or hindering	5.140 (6.315)	6.920 (9.634)	62823.5	.007	−0.318
S9: religion and the supernatural	0.140 (0.738)	0.440 (1.587)	64677.0	.001	−0.324
T1: time	11.3 (12.639)	12.94 (15.022)	67348.0	.16	−0.181
X3: sensory	4.920 (7.606)	2.020 (4.618)	50469.0	<.001	0.684
X9: ability	1.88 (3.619)	1.83 (3.612)	69246.0	.37	0.019
Z2: geographical names	0.550 (1.496)	3.120 (6.184)	45505.5	<.001	−0.674
Z6: negative	5.840 (5.958)	3.080 (4.861)	51392.0	<.001	0.764
Z8: pronoun	59.79 (53.287)	31.56 (38.830)	46155.0	<.001	0.907
Z99: unmatched expressions	13.74 (17.037)	37.58 (49.684)	39069.0	<.001	−0.776

^a^Asymptotic significance (two-tailed).

## Methods

We applied machine learning algorithms to learn the important features for detecting the writing styles of web-based health educational resources on children’s health promotion and education. Recursive feature elimination (RFE), ridge classifier (RC), extreme gradient boosting (XGBoost) [36], and support vector machine (SVM) [37] were used to assist in automatic feature selection. RFE is commonly used with SVM (denoted as RFE_SVM) to build a model and remove unimportant features [38]. In addition to linear models such as SVM, tree-based models are also an effective method to learn feature importance, and XGBoost was used as the learning estimator of RFE (denoted as RFE_XGB). For algorithms RC, SVM, and RFE, we used the implementation in scikit-learn [39]. For XGBoost, we used the Python package xgboost [40].

For the RC and RFE algorithms, scikit-learn has built-in cross-validation variants *RidgeClassifierCV* and *RFECV*, which perform leave-one-out five-fold cross-validation to search for the best hyper-parameters and select the best cross-validated features, respectively. For SVM, which only needs to tune the regularization parameter *C*, we applied the commonly used *GridSearchCV* for hyperparameter tuning. The *GridSearchCV* algorithm performs an exhaustive search over specified parameter values to determine the best and cross-validated parameter values of the model. For XGBoost, which has nine hyper-parameters including some continuous ones, we applied *RandomizedSearchCV*, which performs a randomized search over parameters and samples a fixed number of parameter settings from the specified distribution. We set the number of parameter settings *n_iter* of *Randomized SearchCV* as 300. The hyperparameter *n_iter* defines the number of parameter settings that are sampled. With a large value of *n_iter*, the algorithm was able to find better hyper-parameters from a large parameter setting with high quality. The fine-tuned results of the better hyper-parameters are shown in Multimedia Appendix 2. For the hyper-parameters that were not listed, we used the default values in the model.

**Figure 1:**
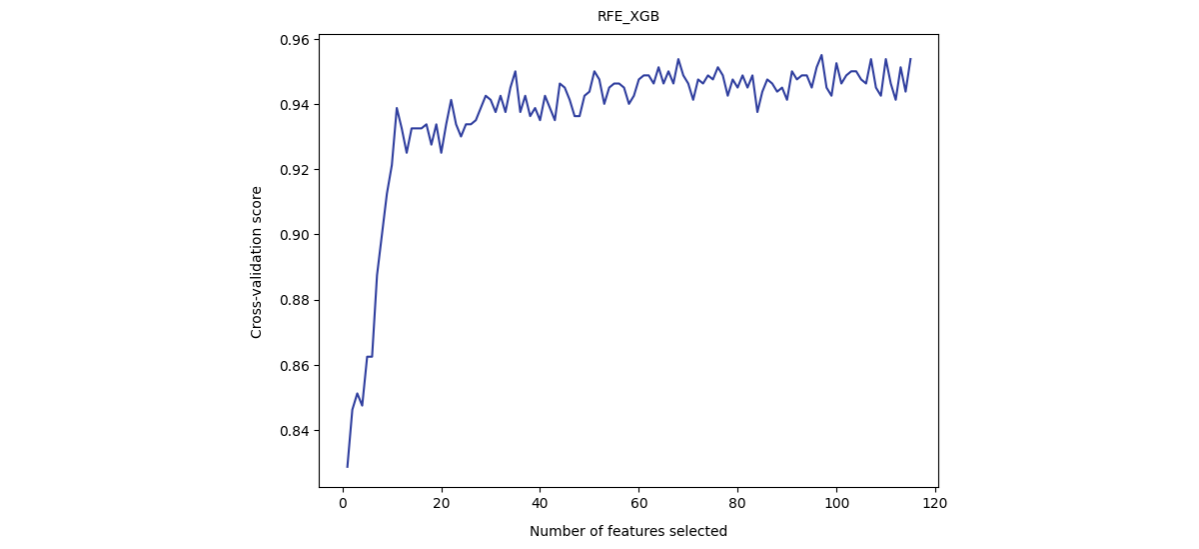
Automatic tuning of the number of features selected with cross-validation of RFE_XGB.

**Figure 2:**
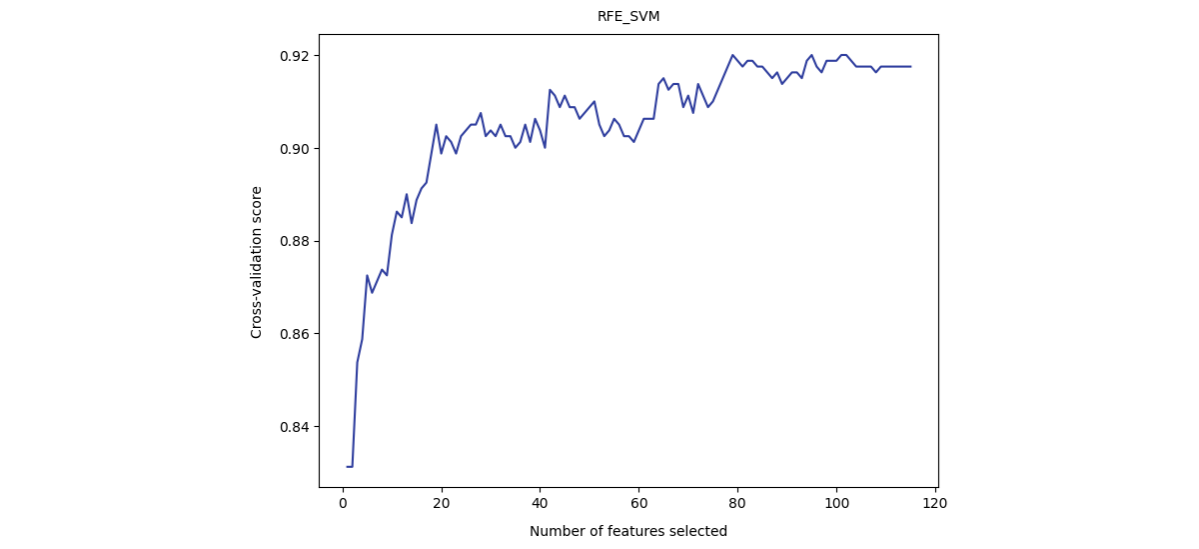
Automatic tuning of the number of features selected with cross-validation of RFE_SVM.

**Figure 3:**
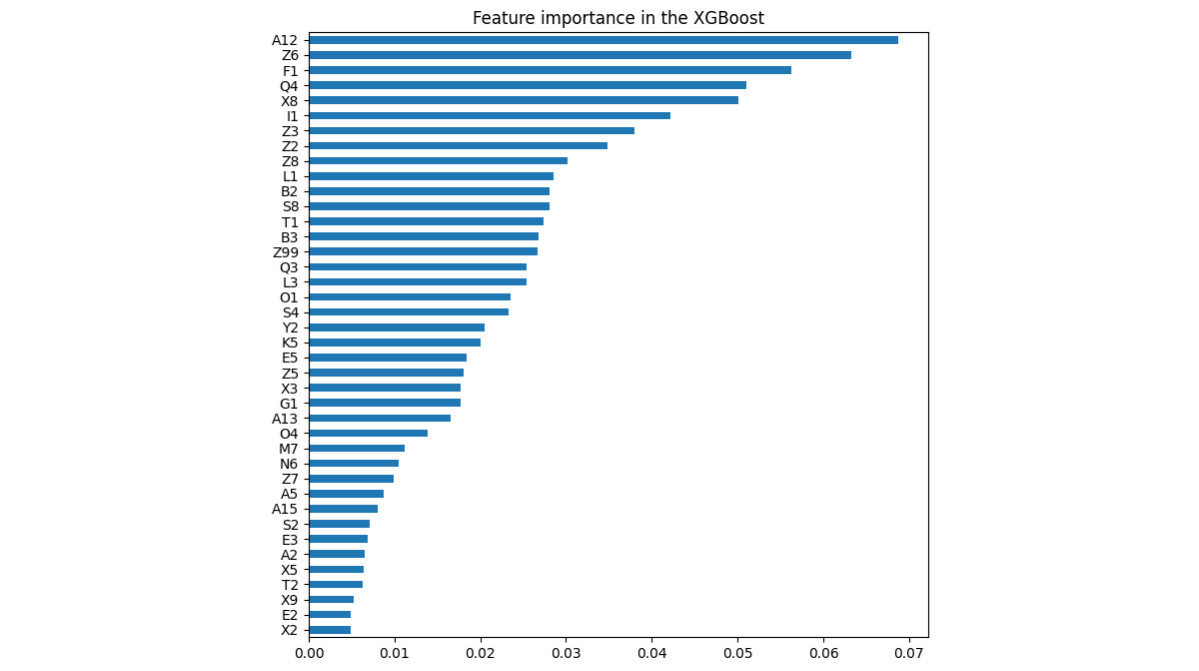
Automatic feature importance ranking using extreme gradient boost tree. XGBoost: extreme gradient boosting.

**Figure 4:**
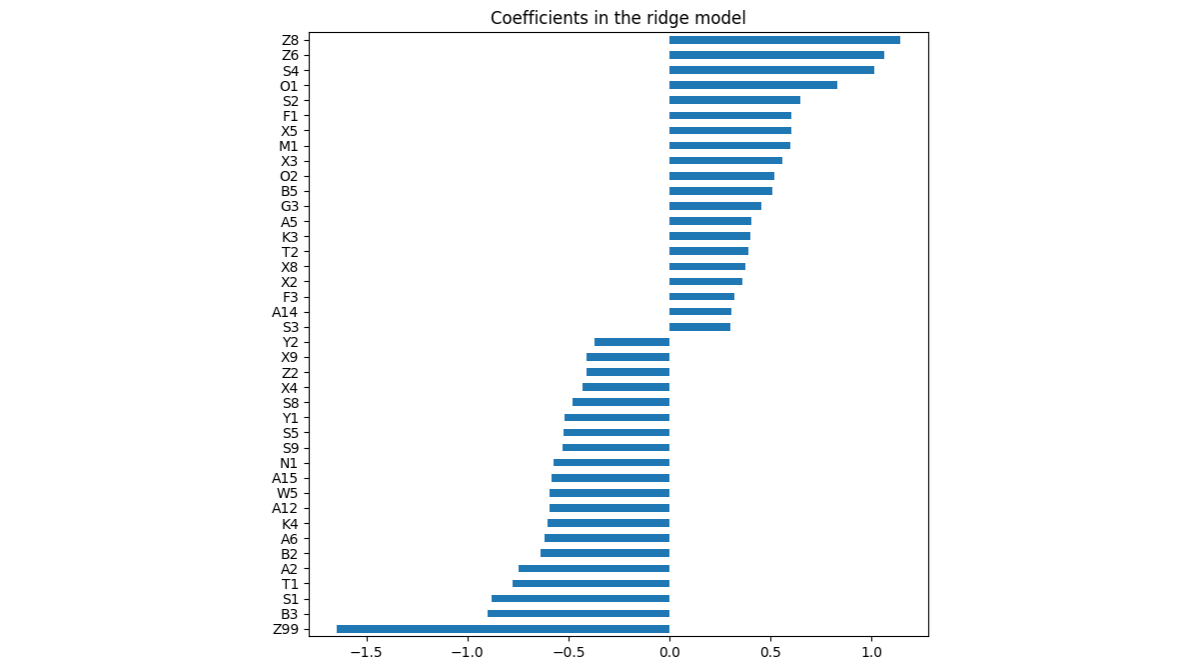
Automatic feature importance ranking using the ridge classifier.

We applied RFE_SVM and RFE_XGB to evaluate the cross-validation score when increasing the number of selected features. The automatic tuning results of the number of features selected by cross-validation are shown in Multimedia Appendix 2. As shown in the results (Figures 1 and 2), both the SVM and XGBoost model gained a nearly stable cross-validation score greater than 0.9 when the number of selected features was equal to or greater than 40. This result indicated that when only 40 features were used, the model was still able to achieve good performance, and adding more features did not help much. As a result, we applied *40* as a threshold to select the top 40 important features learned by RC and XGBoost. The details of the selected top 40 features of RC and XGBoost are shown in Multimedia Appendix 2 and Figures 3 and 4. RFE_SVM learned 95 features, eliminating 20 unimportant features from all 115 features. For the RFE_XGB, 97 features were selected, and 18 unimportant features were eliminated. Finally, the intersected 19 features from the RC, XGBoost, RFE_SVM, and RFE_XGB were selected as automatically learned features from the machine learning algorithms.

## Results

### Feature Selection Results

Table 2 shows the performance of the three machine learning classifiers on the testing data, which were largely similar in terms of overall model accuracy, macro average F1, and F1 for adult- and children-oriented health readings. The top semantic features in the initial automatic feature selection were as follows (for a detailed description of these codes, see the USAS):

- RC: Z99, B3, S1, T1, A2, B2, A6, K4, A12, W5, A15, N1, S9, S5, Y1, S8, X4, Z2, X9, Y2, S3, A14, F3, X2, X8, T2, K3, A5, G3, B5, O2, X3, M1, X5, F1, S2, O1, S4, Z6, Z8
- XGB Tree: X2, E2, X9, T2, X5, A2, E3, S2, A15, A5, Z7, N6, M7, O4, A13, G1, X3, Z5, E5, K5, Y2, S4, O1, L3, Q3, Z99, B3, T1, S8, B2, L1, Z8, Z2, Z3, I1, X8, Q4, F1, Z6, A12
- RFE using SVM as the feature scoring algorithm: A2, A3, A4, A5, A6, A7, A9, A10, A11, A12, A13, A14, A15, B1, B2, B3, B5, C1, E4, E5, E6, F1, F3, F4, G1, G2, G3, H2, H3, H4, H5, I1, I2, I3, I4, K1, K2, K3, K4, K5, L1, L2, L3, M1, M3, M4, M5, M6, M8, N1, N2, N3, N4, N5, N6, O2, O3, O4, P1, Q1, Q2, Q3, Q4, S1, S2, S3, S4, S5, S6, S7, S8, S9, T1, T2, T4, W1, W2, W4, W5, X2, X3, X4, X5, X6, X7, X9, Y1, Y2, Z1, Z2, Z3, Z4, Z6, Z8, Z99
- RFE using XGB as the feature scoring algorithm: A1, A2, A3, A4, A5, A6, A7, A8, A9, A10, A11, A12, A13, A14, A15, B1, B2, B3, B4, B5, C1, E1, E2, E3, E4, E6, F1, F2, G1, H3, H4, I1, I2, I3, I4, K3, K4, K5, K6, L2, L3, M1, M2, M3, M4, M5, M6, M7, N1, N3, N4, N5, N6, O1, O2, O3, O4, Q1, Q2, Q3, Q4, S1, S2, S3, S4, S5, S6, S7, S8, S9, T1, T2, T3, T4, W1, W3, W4, X2, X3, X5, X6, X7, X8, X9, Y1, Y2, Z0, Z1, Z2, Z3, Z4, Z5, Z6, Z7, Z8, Z9, Z99
- The common 19 features of the four feature selection algorithms are as follows: Z8, S2, S8, F1, A5, S4, X3, M1, T1, S5, S9, Z99, A15, S1, X9, Z6, B2, Z2, B3.

**Table 2:** Classifiers used for automatic feature selection.

Classifier and text class			Accuracy		Macro average F1^a^		Precision		Recall		F1
**Ridge classifier**			0.925		0.89						
	Adult-oriented readings					0.99		0.91		0.95	
	Children-oriented readings					0.74		0.97		0.84	
**SVM^b^**			0.93		0.89						
	Adult-oriented readings					0.95		0.96		0.96	
	Children-oriented readings					0.84		0.8		0.82	
**XGB^c^**			0.94		0.90						
	Adult-oriented readings					0.95		0.98		0.96	
	Children-oriented readings					0.91		0.78		0.84	

^a^F1 = 2 × [(precision × recall) / (precision + recall)].

^b^SVM: support vector machine.

^c^XGB: extreme gradient boosting.

Table 3 shows the comparison of the performance of algorithms using the original 115 features as predictor variables and the automatically selected 19 semantic features as predictor variables. With GNB classifier, we reduced the predictor variables from 115 to 19, the mean sensitivity (of the five folds of data) decreased from 0.685 to 0.634 (0.074%), the mean specificity increased from 0.771 to 0.903 (17.04%), and the mean area under the receiver operating characteristic curve (AUC) increased from 0.822 to 9.882 (7.21%). Similar patterns were observed with K-nearest neighbor (KNN). Mean sensitivity decreased from 0.973 to 0.943 when the predictor variables reduced in number. By contrast, the model mean specificity increased by 33.7% from 0.526 to 0.703 and the mean AUC increased by 3.79% from 0.901 to 0.935. This suggested that for some algorithms such as GNB and KNN, feature selection can increase the model efficiency, at least partially. However, with XGB, both mean sensitivity and mean specificity decreased by around 0.5%, resulting in a decrease of mean AUC of 0.95%. The decrease in the mean sensitivity, mean specificity, and mean AUC of XGB and the decrease in mean specificity of GNB and KNN using automatically selected features indicated that further linguistic revision was needed. Linguistic review of the automatically selected features will ascertain whether the automatically selected features were linguistically meaningful and explainable.

Features that were deemed linguistically irrelevant or unexplainable will be replaced by semantic features that are highly relevant and significant for health language studies. Incorporating insights from language studies into automatic feature selection will help in the development of adaptive and interpretable machine learning algorithms. Increasing the interpretability and practical usability of algorithms can be achieved at the stage of the linguistic review of automatically selected feature sets.

We eliminated S9, T1, S2, and Z2 and added X8, A12, A11, A13, and A14. These were the semantic features that were highly relevant to health linguistics. X8 are terms depicting the level of effort and resolution. This is a statistically significant feature of children’s educational resources (*P*<.001; Cohen *d*=0.803). Typical words of X8 were tried, fights, hard, fighting, try, and struggling, which were prevalent in health educational resources for children to describe bodily reactions to diseases and viruses. In contrast, adult-oriented health education resources were abundant in words and expressions of A12, which were abstract terms denoting the varying levels of difficulties: challenge, adversity, and complexity. The independent *t* test showed that A12 was a characteristic semantic feature of general health materials (*P*<.001; Cohen *d*=−0.234). A11 included abstract terms denoting importance or significance and abstract terms denoting noticeability or markedness. Typical words of A11 were main, significant, important, serious, principal, emergency, distinctive, urgent, crucial, and emergencies that were abundant in adult health educational resources (*P*<.001; Cohen *d*=−0.0348). A13 included words such as maximizers, boosters, approximators, and compromisers (*P*<.001; Cohen *d*=0.645). Typical words of A13 were very, almost, more, as, about, up, to, approximately, fully, even, and enormously, which were prevalent in children’s health education resources. Finally, A14 focused on subjuncts that drew attention to or to focus upon (*P*=.04; Cohen *d*=0.519). Typical words of A14 were especially, just, and only, which were highly frequent in children’s health educational readings.

**Table 3:** Performance of classifiers using 115 (originally tagged) and 19 (automatically selected) features.

Classifier and feature sets		Sensitivity, mean (SD)	Specificity, mean (SD)	AUC^a^, mean (SD)
**GNB^b^**				
	All 115 features	0.685 (0.125)	0.771 (0.116)	0.822 (0.062)
	Automatically selected 19 features	0.634 (0.074)	0.903 (0.063)	0.882 (0.054)
**KNN^c^**				
	All 115 features	0.973 (0.013)	0.526 (0.096)	0.901 (0.032)
	Automatically selected 19 features	0.943 (0.028)	0.703 (0.048)	0.935 (0.023)
**XGB^d^**				
	All 115 features	0.982 (0.01)	0.766 (0.059)	0.978 (0.012)
	Automatically selected 19 features	0.970 (0.019)	0.737 (0.051)	0.970 (0.016)

^a^AUC: area under the receiver operating characteristic curve.

^b^GNB: Gaussian Naive Bayes.

^c^KNN: K-nearest neighbor algorithm.

^d^XGB: extreme gradient boosting.

Table 4 shows the linguistic profiling framework we developed for the revised set of semantic features. It includes the 15 automatically selected features and the manually added five features based on their relevance for health linguistic and language studies, as well as their function as statistically significant, large characteristic features of children- versus adult-oriented health educational readings. The linguistic framework for comparing health texts intended for these two distinct readerships contained three key dimensions that were cognitive abilities, social context of health issues, and user-adaptive health communication style. Under each dimension, there were several contrastive semantic features which help to distinguish health readings for different readers. Within the dimension of cognitive abilities, four semantic features reflect the different scope of health knowledge of children versus adults. For example, F1 food-related words and expressions (creams, peanuts, spread, appetite, foods, salt, sugar, meal, pasta, and rice), and X3 sensory expressions describing taste, color, sight, feel, and sound of things (hearing, see, notice, scented, hear, watch, sound, smell, colorful, etc) were prevalent in children’s health readings as their main approach to health knowledge acquisition. In contrast, more abstract, complex, rare, difficult words were characteristic features of adult health readings—B2: medicine (medical, condition, disorder, stroke, tumor, injury, illness, health, miscarriage, infertility, etc); B3: medical treatment (neurological, diagnosed, computed tomography, cure, scan, medicinal, analgesic, healing, diagnosis, drugs, etc), and Z99: complex, out-of-dictionary words (cyclones, aldosterone, noncancerous, vestibulocochlear, neurofibromatosis, tinnitus, muskrat, ondatra, zibethicus, herbivore, alkanes, esters, aldehydes, etc).

Children and adults also use different approaches to assess health events and situations: A5 words that evaluate events in terms of good or bad and false or true were more prevalent in children’s readings with typical words such as wrong, right, better, good, true, positive, improved, greater, ok, and best. In contrast, A15 words that assess health situations in terms of safety, risk, and harm were more prevalent in adult health readings with typical expressions that we found in the corpus: at-risk, safe, dangerous, exposures, hazard, safety, insurance, warning, alert, and alarming. X9 terms describing success and failure, gains and losses, and benefits and risks were also prevalent in adult health materials. This finding aligns well with the latest research on health communication using the Prospect Theory [41], which highlights the human propensity to maximize benefits and minimize risks, including in health care and medical settings. Typical words of X9 included effective, successful, lose, achieve, gains, go wrong, overcome, solve, cope, and competent. The complexity of actions is another important feature of health education reading [42,43]. In children’s health readings, simple actions and verbs describing the direction of movements were prevalent—typical words in M1 were moving, coming, and going, get, follow, step, and steps. In contrast, the mean frequency of S8 words describing levels of help, obstacles, and hindrance was statistically higher in adult health readings such as stop, prevent, cooperate, benefits, resistance, protect, protecting, support, supporting, and help.

We also identified predictor features that are relevant to the social context of health issues [44]. This dimension includes two sets of semantic features of interpersonal relations and the socioeconomic contexts of health issues. For example, S4 words of kinships (family, parents, siblings, relatives, children, household, families, etc) were more common in children’s health readings, whereas S5 words of people’s social groups and affiliation were prevalent in adult health educational readings such as network, loneliness, community, member, partnership, and alliance. Another important semantic feature is S1 terms related to participation, involvement, entitlement, and eligibility or describing personality traits such as strength, weakness, vulnerability, and disadvantaged. Typical words of S1 were vulnerable, self-esteem, meeting, helplessness, social, and contacts, which were highly frequent in adult health readings. We could not find an equivalent semantic feature class in children’s health readings to match S1 as a characteristic of adult health readings.

The health communicative style is another key dimension of semantic features [30]. We found that an effective communicative style is particularly relevant for children-oriented health educational readings [45]. For example, to match the machine learning–selected feature of A11 terms describing importance and priority, we added two functionally equivalent semantic features that were prevalent in children’s health readings to help increase the emphasis and stress on the key health messages of the texts: A13 and A14. Both were mostly adverbs describing the degree, levels, extent, severity of objects, and events. For example, typical words in A13 were very, almost, more, as, about, up, to, approximately, fully, even, enormously; and typical words of A14 were especially, just, and only. These words stand in contrast with A11 words that characterize the prioritization and importance attribution among adults: main, significant, important, serious, principal, emergency, distinctive, urgent, crucial, and emergencies. Finally, terms that help increase the logical coherence of health readings were highly frequent in children’s health readings but not in adult readings. These include Z8, the use of pronouns (it, this, who, that, you, what, we, they, their, which, your, our, and anything), and Z6, the use of negative expressions.

**Table 4:** Revised linguistic evaluation framework with final 20 features.

Dimensions of linguistic analyses		Texts on children’s health	Texts on adults’ health
**Cognitive abilities**			
	Scope of health knowledge	F1 (food) X3 (sensory: taste, sound, and touch)	B2 (medicine); B3 (medical treatment) Z99 (complex and out-of-dictionary words)
	Assessment of situations	A5 (good or bad and true or false)	A15 (safety or danger) X9 (success or failure, gains or loss, and benefits or risks)
	Describing efforts	X8 (level of efforts or resolution)	A12 (level of difficulty)
	Complexity of actions	M1 (actions of movement)	S8 (level of help or hindrance)
**The social context of health issues**			
	Interpersonal relations	S4 (kin)	S5 (social groups and affiliation)
	Socioeconomic context	N/A^a^	S1 (terms related to participation, involvement, entitlement, eligibility; or describing personality traits such as strength, weakness, vulnerability, and disadvantaged)
**Communicative style**			
	Attention emphasis and stress	A13 (degree) A14 (particularizers)	A11 (importance)
	Logical coherence	Z8 (pronouns) Z6 (negative)	N/A

^a^N/A: not applicable.

Table 5 shows features in the linguistic evaluation framework for a binary logistic regression analysis (enter) with children-oriented health resources as the reference class. The statistical result aligns with the linguistic analysis well: 10 semantic features had negative unstandardized coefficients and less than 1 odds ratio, suggesting that with the increase of values in these features, the odds of the health text being a children-oriented health reading were higher than those of the health text being an adult health reading. For example, the odds ratio of Z6 negative expressions (*P*<.001) was 0.778 (95% CI 0.69-0.876), which means that with the increase of one Z6 word, the odds of the health text being an adult health reading reduced by a mean of 22.2%. The odds ratio of S4 (words describing kinships; *P*<.001) was 0.823 (95% CI 0.746-0.907), meaning with the increase of one word of S4 (such as parents, siblings, grandparents, etc), the odds of the health text being a children’s reading was 17.7% higher than those of the health text being an adult-oriented health reading. X8 (*P*=.07), A14 (*P*=.66), M1 (*P*=.17), and A13 (*P*=.39) were statistically insignificant predictor variables. Similarly, 10 semantic features were identified as characteristic features of adult health readings: A11, B2, B3, Z99, X9, S8, S5, S1, A12, A15. A11 and X9 were statistically insignificant predictor variables. The odds ratio of A15 was 1.945 (95% CI 1.335-2.833), which means that with the increase of one word of A15 (words evaluating safety, danger, or risks of health events), the odds of the health text being an adult reading was 94.5% higher than those of the text being a children-oriented health reading.

**Table 5:** Predictor variables of binary logistic regression (children=0; adult=1).

Relevance of semantic features to outcomes			Values						
			β (SE)		Wald test		*P* value		OR^a^ (95% CI)
**Semantic features related to higher ORs of health texts on children’s health**									
	Z6	−0.252 (0.061)		16.966		<.001		0.778 (0.690-0.876)	
	X8	−0.228 (0.127)		3.233		.07		0.796 (0.621-1.021)	
	S4	−0.195 (0.050)		15.351		<.001		0.823 (0.746-0.907)	
	X3	−0.134 (0.033)		16.715		<.001		0.875 (0.820-0.933)	
	A5	−0.104 (0.038)		7.418		.006		0.902 (0.837-0.971)	
	A14	−0.063 (0.144)		0.192		.66		0.939 (0.707-1.246)	
	M1	−0.054 (0.039)		1.927		.17		0.948 (0.878-1.022)	
	F1	−0.038 (0.011)		11.374		.001		0.963 (0.942-0.984)	
	A13	−0.036 (0.042)		0.744		.39		0.965 (0.889-1.047)	
	Z8	−0.021 (0.008)		7.589		.006		0.979 (0.964-0.994)	
**Semantic features related to higher ORs of health texts on adults’ health**									
	A11	0.030 (0.086)		0.124		.73		1.031 (0.871-1.219)	
	B2	0.032 (0.013)		6.397		.01		1.032 (1.007-1.058)	
	B3	0.066 (0.019)		12.425		<.001		1.068 (1.030-1.108)	
	Z99	0.067 (0.011)		35.849		<.001		1.069 (1.046-1.093)	
	X9	0.118 (0.064)		3.400		.07		1.126 (0.993-1.277)	
	S8	0.162 (0.040)		16.137		<.001		1.176 (1.087-1.273)	
	S5	0.248 (0.057)		19.056		<.001		1.281 (1.146-1.432)	
	S1	0.279 (0.085)		10.703		.001		1.322 (1.118-1.562)	
	A12	0.297 (0.102)		8.573		.003		1.346 (1.103-1.642)	
	A15	0.665 (0.192)		12.003		.001		1.945 (1.335-2.833)	

^a^OR: odds ratio.

### Performance Comparison of Classifiers Using Three Sets of Features

Tables 6-10 show the results of the comparison of GNB algorithms developed using the originally tagged multidimensional feature set (n=115), automatically selected feature set (n=19), and linguistically enhanced feature set (n=20). Table 7 shows that both the automatically selected and the linguistically enhanced feature set achieved statistically improved AUC over the original high-dimensional feature set: automatically selected (*P*=.008) and linguistically enhanced (*P*=.02), significant at the adjusted *P*=.17 using Bonferroni correction. The difference in AUC between the two streamlined feature sets was not statistically significant (*P*=.56). In terms of model sensitivity, the automatically selected feature set did not achieve statistically significant improvement over the OR feature set (*P*=.13) but the linguistically enhanced feature set did (*P*=.01). The sensitivity of the linguistically enhanced feature set was also statistically improved over the automatically selected feature set (*P*<.001). In terms of model specificity, the automatically selected feature set did not improve over the OR feature set (*P*=.10), but the linguistically enhanced feature set did (*P*=.01). The specificity between the automatically selected and linguistically enhanced feature sets did not differ significantly (*P*=.53). Finally, in terms of macro F1, which provides a balanced assessment of the model performance, the automatically selected feature set did not improve over the baseline OR feature set (*P*=.98). The linguistically enhanced feature set improved significantly over the OR feature set (*P*=.006) and automatically selected feature set (*P*=.001).

**Table 6:** Performance of machine learning models using different sets of features as predictors.

Algorithm	AUC^a^, mean (SD)	Sensitivity, mean (SD)	Specificity, mean (SD)	Macro F1^b^, mean (SD)
115 features	0.8224 (0.0617)	0.6848 (0.1252)	0.7714 (0.1161)	0.6336 (0.080)
19 features (automatic selection)	0.8817 (0.0539)	0.6339 (0.0743)	0.9029 (0.0626)	0.6333 (0.067)
20 features (linguistic review)	0.8888 (0.0315)	0.7733 (0.076)	0.8629 (0.0843)	0.7248 (0.0451)

^a^AUC: area under the receiver operating characteristic curve.

^b^F1 = 2 × [(precision × recall) / (precision + recall)].

**Table 7:** Pairwise corrected resampled *t* test of area under the receiver operating characteristic curve differences using three sets of features as predictors.

Pair	Description	Mean difference (%)	95% CI of mean difference	*P* value (two-tailed)
1	19 features versus 115 features	7.2096	0.0059 to 0.1126	.008^a^
2	20 features versus 115 features	8.0729	−0.0071 to 0.1399	.02^a^
3	20 features versus 19 features	0.8052	−0.0421 to 0.0563	.56

^a^*P* value significant at .0167 (Bonferroni correction).

**Table 8:** Pairwise corrected resampled *t* test of sensitivity differences using three sets of features as predictors.

Pair	Description	Mean difference (%)	95% CI of the mean difference	*P* value (two-tailed)
1	19 features versus 115 features	−7.4336	−0.1699 to 0.0681	.13
2	20 features versus 115 features	12.9204	−0.016 to 0.1929	.011^a^
3	20 features versus 19 features	21.9885	0.1048 to 0.174	<.001^a^

^a^*P* value significant at .0167 (Bonferroni correction).

**Table 9:** Pairwise corrected resampled *t* test of specificity differences using three sets of features as predictors.

Pair	Description	Mean difference (%)	95% CI of the mean difference	*P* value (two-tailed)
1	19 features versus 115 features	17.037	−0.1389 to 0.4017	.10
2	20 features versus 115 features	11.8519	−0.0163 to 0.1991	.01^a^
3	20 features versus 19 features	−4.4304	−0.2923 to 0.2123	.53

^a^*P* value significant at .0167 (Bonferroni correction).

**Table 10:** Pairwise corrected resampled *t* test of macro F1 differences using three sets of features as predictors.

Pair	Description	Mean difference (standardized; %)	95% CI of the mean difference	*P* value (two-tailed)
1	19 features versus 115 features	−0.0539	−0.0555 to 0.0548	.98
2	20 features versus 115 features	14.3813	0.0158 to 0.1665	.006^a^
3	20 features versus 19 features	14.4430	0.0422 to 0.1407	.001

^a^*P* value significant at .0167 (Bonferroni correction).

**Figure 5:**
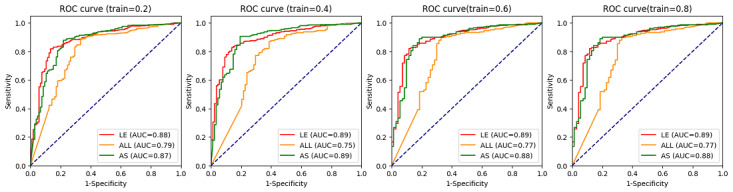
Scalability and effectiveness of the 20 linguistically enhanced features. AS: automatically selected; AUC: area under the receiver operating characteristic curve; LE: linguistically enhanced; ROC: receiver operating characteristic curve.

We also tested the scalability and effectiveness of the 20 linguistically enhanced features (Figure 5). We compared the performance with 115 initial all features (ALL) and 19 automatically selected features. The data were randomly divided into a training set and test set with different split rates of 0.2, 0.4, 0.6, and 0.8. The performance was evaluated using receiver operating characteristic curve and AUC metrics. As shown in Figure 5, the model using linguistically enhanced features always yielded the best performance with a stable AUC score of 0.89 with the different training data set size. Moreover, when using only 20% data for training (train=0.2), the model using linguistically enhanced features still achieved a much higher performance than the baseline (using ALL features), demonstrating its effectiveness and potential for scalability. Thus, incorporating both linguistic features and machine learning features can better help in the interpretation and auto-learning of health educational materials.

## Discussion

### Principal Findings

Our study illustrated machine learning–assisted selection of textual features to develop new algorithms to predict the content and writing style of credible web-based resources for children’s health education and promotion among the parents and caregivers of young children. We used high-quality health educational resources developed by influential children’s health promotion and educational organizations as training data. We illustrated that feature selection to reduce high-dimensional feature sets is an effective method for improving the efficiency of machine learning algorithms, as shown by the improved performance of the AUC of the model using automatically selected features (n=19) as predictor variables over the originally tagged feature set (n=115; *P*=.008). However, specificity, sensitivity, and macro F1 did not improve when using the automatically selected feature set. We then refined automatic feature selection by incorporating linguistic insights from health linguistics and user-oriented health communication. The linguistically enhanced features led to a statistically significant improvement in sensitivity; macro F1 over the automatically selected feature set: sensitivity (*P*<.001) and macro F1 (*P*=.001); and statistically significant improvement of AUC, sensitivity, specificity, and macro F1 over the original high-dimensional feature set: AUC (*P*=.02), sensitivity (*P*=.01), specificity (*P*=.01), and macro F1 (*P*=.006).

Machine learning algorithms were known for their lack of interpretability. Through the successive permutation of the linguistically enhanced predictor variables in the developed GNB algorithm, we explored the individual impact of each feature on the model’s sensitivity and specificity. Two sets of semantic features emerged as large contributors to the model’s ability to predict the suitability of health educational resources for adults and children, respectively. We found the final algorithm interpretable using the linguistic profiling framework developed for those automatically selected features. For the prediction of adult-oriented health education readings, that is, features highly relevant for the sensitivity of the model, 11 semantic features were identified as large contributors as indicated by the decrease of sensitivity in their absence: X3 (−9.4%; words of sensory: taste, sound, touch, sight, smell, etc), S4 (−8.93%; kinships), Z99 (−8.78%; complex words), A14 (−7.99%; focusing subjuncts that draw attention to or to focus upon), Z8 (−6.9%) (pronouns), A11 (−6.11%; terms describing importance and priority), S1 (−5.96%; terms of participation, involvement, entitlement, and eligibility or describing personality traits such as strength, weakness, vulnerability, and disadvantaged), A5 (−5.94%; words of evaluating good or bad or true or false), B3 (−5.33%; medical treatment), S8 (−4.86%; words describing levels of help, obstacles, and hindrance), X9 (−0.31%; success or failure; gains or loss; or benefits or risks).

**Figure 6:**
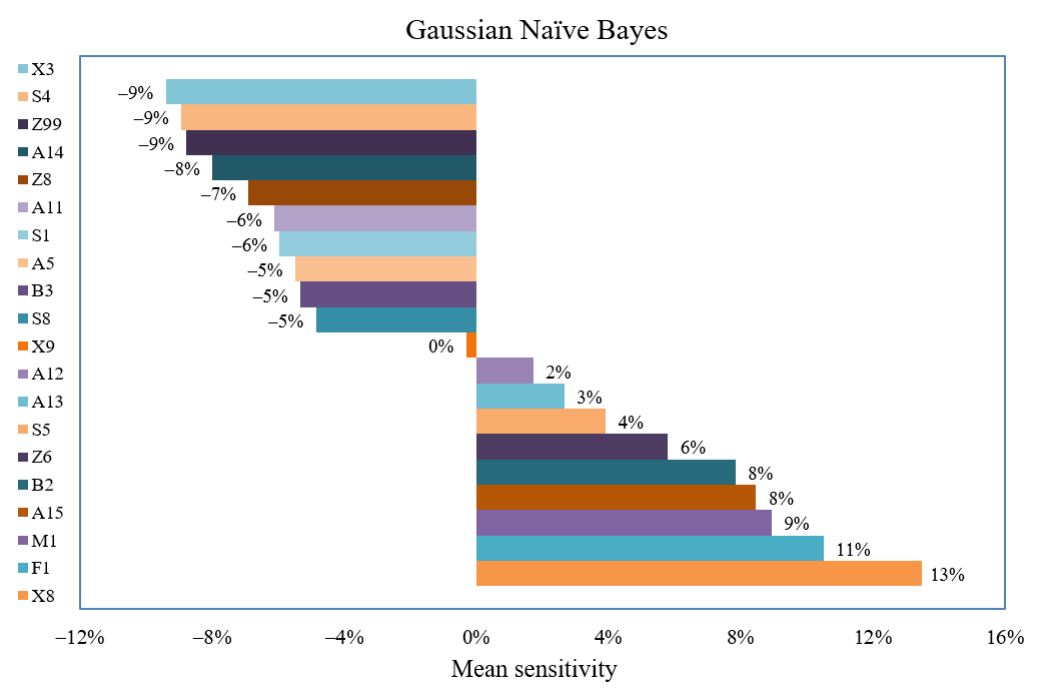
Impact of selected features on mean sensitivity.

For the prediction of health education readings on children’s health, that is, features highly relevant for the specificity of the model, 10 semantic features were identified as large contributors, as shown by the decrease in model specificity with the successive permutation of these features (Figure 6): X8 (−24.5%; words describing efforts and resolution), F1 (−23.18%; food-related words), S5 (−14.57%; social groups and affiliation), A15 (−9.93%; words evaluating safety and danger), M1 (−9.27%; movement words), B2 (−9.27%; medicine), Z6 (−8.61%; negative), A13 (−5.96%; degree), A12 (−2.65%; difficulty), and X9 (−0.66%; success or failure; gains or loss; and benefits or risks).

**Figure 7:**
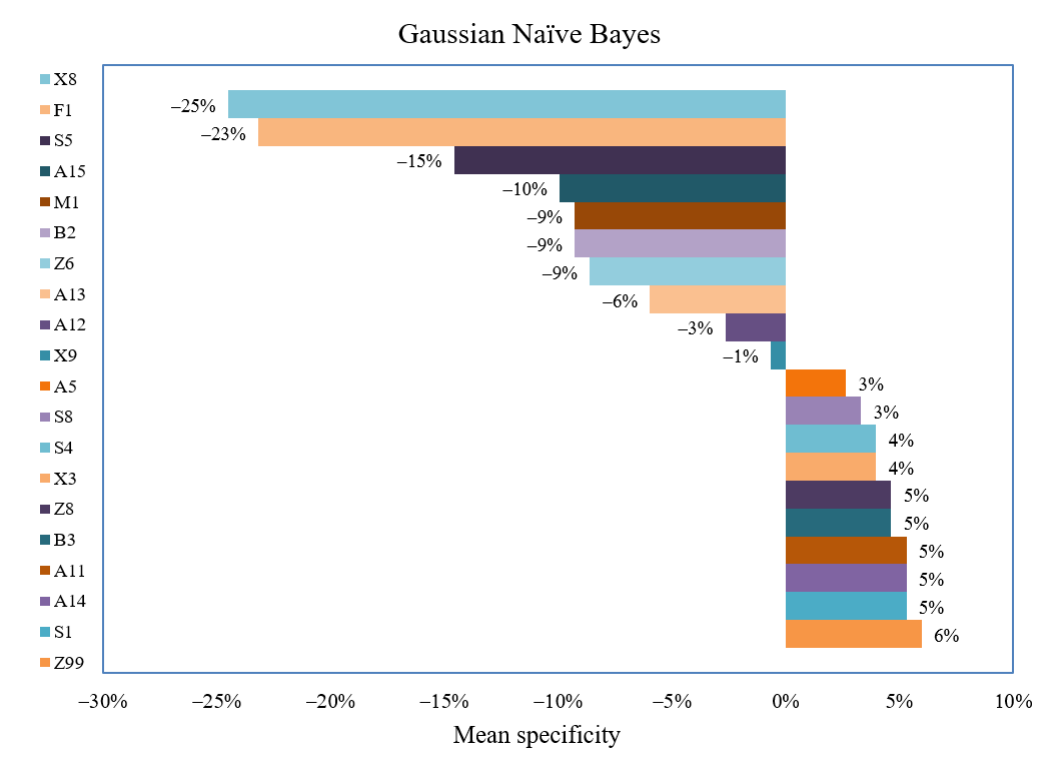
Impact of selected features on mean specificity.

It is worth noting that features identified as key contributors to model sensitivity were not necessarily features that were statistically significant in adult-oriented health readings (Table 1). For example, X3, S4, A14, Z8, and A5 were statistically significant in children’s health resources, which however had large impacts on the model sensitivity (Figure 7). Similarly, S5, A15, B2, A12, and X9 were statistically significant features of adult health materials but they also had an impact on model specificity, which is the ability of the machine learning algorithm to predict health texts as children-oriented health materials. This led to our interpretation that the newly developed algorithm represents a balanced mix of linguistically relevant, meaningful semantic features that were statistically significant in either children or adult health materials. Thus, the approach to outcome prediction of machine learning differs significantly from that of statistical analysis. However, our study demonstrated that both statistical and linguistic insights can improve the performance of machine learning–assisted feature selection and subsequent prediction.

### Limitations and Future Research

The size of the training data set was relatively small, with a couple hundred texts of children-oriented health readings. However, this reflects the reality, as children’s health educational resources are much less than adult health readings. As a result, the model specificity was consistently lower than the model sensitivity. In addition, in the linguistic evaluation framework (Table 4), the structure was not well balanced. Items were not complete for all evaluation subcategories, such as health communication styles. Further studies are required to fill the research gaps that emerged in this study.

### Conclusions

Our study has shown that children-oriented and adult-oriented health educational readings in English have distinct semantic features that can be effectively exploited to develop machine learning algorithms with proven discriminatory accuracy. Specifically, we identified three large sets of semantic features related to the varying cognitive approaches to health information acquisition, the social contexts of health issues, and user-adaptive health communication styles. Machine learning is known to lack interpretability. Our study developed algorithms that are interpretable from the perspective of linguistics and user-oriented health information assessment. Thus, our study shows that a more integrated approach to computerized health information assessment combining insights from fields such as linguistics and health education can help harness the power of machine learning to advance applied social and health research.

## Appendix

Multimedia Appendix 1 Health on the Net Foundation–certified websites used.

Multimedia Appendix 2 Core parameters of the hyper-parameter tuning of ridge classifier, extreme gradient boosting, support vector machine, and recursive feature elimination.

## Abbreviations

AUC: area under the receiver operating characteristic curve
GNB: Gaussian Naive Bayes
KNN: K-nearest neighbor
PEMAT: Patient Education Materials Assessment Tool
RC: ridge classifier
RFE: recursive feature elimination
SVM: support vector machine
USAS: University of Lancaster Semantic Annotation System
XGBoost: extreme gradient boosting
