# Supplementary material for: Predicting Writing Styles of Web-Based Materials for Children’s Health Education Using the Selection of Semantic Features: Machine Learning Approach
Source: JMIR Med Inform. 2021 Jul 22;9(7):e30115. doi: 10.2196/30115 (PMC8367110; doi:10.2196/30115)
Supplement: Multimedia Appendix 1 [file medinform_v9i7e30115_app1.docx]

**Appendix 1 HON.Net certified websites used**

www.mayoclinic.org

www.betterhealth.vic.gov.au

www.who.int

www.healthdirect.gov.au

https://www.webmd.com/

https://www.healthline.com/

https://www.verywellhealth.com/

https://www.everydayhealth.com/

https://medlineplus.gov/
