# Supplementary material for: Predicting Writing Styles of Web-Based Materials for Children’s Health Education Using the Selection of Semantic Features: Machine Learning Approach
Source: JMIR Med Inform. 2021 Jul 22;9(7):e30115. doi: 10.2196/30115 (PMC8367110; doi:10.2196/30115)
Supplement: Multimedia Appendix 2 [file medinform_v9i7e30115_app2.docx]

**Appendix 2 Core parameters of the hyperparameter tuning of Ridge Classifier, XGBoost, SVM and Recursive Feature Elimination**

| Algorithm | Hyper-parameter name | Description | Value |
| --- | --- | --- | --- |
| Ridge Classifier CV | alpha | Regularization strength | 2.9470517025518097 |
| SVM | C | Regularization parameter. The strength of the regularization is inversely proportional to C. | 32.90344562312671 |
|  | kernel | The kernel type to be used in the algorithm. | linear |
|  | probability | Whether to enable probability estimates. | True |
| XGBoost | subsample | Subsample ratio of the training instances. | 0.8894736842105263 |
|  | scale_pos_weight | Control the balance of positive and negative weights, useful for unbalanced classes. | 1 |
|  | reg_lambda | L2 regularization term on weights. | 0.05263157894736842 |
|  | reg_alpha | L1 regularization term on weights. | 0.47368421052631576 |
|  | n_estimators | Number of boosted trees to fit | 116 |
|  | min_child_weight | Minimum sum of instance weight (hessian) needed in a child. | 2 |
|  | max_depth | Maximum depth of a tree. | 2 |
|  | learning_rate | Step size shrinkage used in update to prevent overfitting. | 0.42894736842105263 |
|  | colsample_bytree | The subsample ratio of columns when constructing each tree. | 0.7666666666666666 |
| RFECV | estimator | A supervised learning estimator with a fit method that provides information about feature importance. | SVM or XGBoost |
|  | min_features_to_select | The minimum number of features to be selected. | 1 |
|  | step | the number of features to remove at each iteration. | 1 |
